# Factors Associated with Consumption of Diabetic Diet among Type 2 Diabetic Subjects from Ahmedabad, Western India

**DOI:** 10.3329/jhpn.v30i4.13328

**Published:** 2012-12

**Authors:** Mayur Patel, Ina M. Patel, Yash M. Patel, Suresh K. Rathi

**Affiliations:** ^1^Swasthya Hospital, All India Institute of Diabetes and Research, Narainpura, Ahmedabad 380013, Gujarat, India;; ^2^Department of Community Medicine, SBKS Medical Institute and Research Centre, Sumandeep Vidyapeeth, Piparia, Vadodara 391 760, Gujarat, India

**Keywords:** Cross-sectional study, Diet, Glycaemic control, Glycosylated haemoglobin, Obesity, Type 2 diabetes mellitus, India

## Abstract

This cross-sectional study assessed the current situation of and factors associated with consumption of diabetic diet among 399 type 2 diabetes mellitus (T2DM) subjects from Ahmedabad, Western India. The study was performed with diagnosed (at least one year old) diabetic subjects who attended the Department of Diabetology, All India Institute of Diabetes and Research and Yash Diabetes Specialties Centre (Swasthya Hospital), Ahmedabad during July 2010–November 2010. The subjects completed an interviewer-administered questionnaire. The questionnaire included variables, such as sociodemographic factors, family history of diabetes, behavioural profile, risk profile (glycaemic status, hypertension, and obesity), and diet-related history (consumption of diabetic diet, consumption of low fat/skimmed milk, method of cooking, and sources for diet advice). Blood pressure, body mass index, glycosylated haemoglobin (HbA1c) level, and fasting lipid profile were measured. All analyses including multivariate logistic regression were conducted using SPSS, version 11.5. In total, 399 T2DM subjects (65% male, 35% female) with mean age of 53.16±7.95 years were studied. Although 73% of T2DM subjects were consuming diabetic diet, the good glycaemic control (HbA1c level <7%) was achieved only in 35% of the subjects. The majority (75%) of the subjects had a positive family history of diabetes, and 52% were obese. In 77%, the main source of dietary advice was doctor. In 36%, the main methods of cooking were: boiling and roasting. The final multivariate model showed that visit to dietician, level of education, intake of low fat, and family history of diabetes were independent predictors for diabetic diet consumption among T2DM subjects. However, longitudinal and cohort studies are required to establish the association between consumption of diabetic diet and glycaemic control.

## INTRODUCTION

Type 2 diabetes mellitus (T2DM) is a chronic disease associated with high morbidity and mortality worldwide (1), and India is no exception (2,3). Currently, India is facing a three-fold rise in the prevalence of diabetes in urban as well as in rural area (4). Subjects with T2DM are at high risk of developing micro-vascular and macro-vascular complications; hence, the need for preventive action is widely acknowledged (5). The fundamentals of diabetes control largely depend upon drug therapy and lifestyle measures (increased physical activity and restriction of energy intake/diabetic diet) (6). Improved glycaemic control may reduce the development and progression of diabetic complications to some extent (7). Wealth of information is available on improving glycaemic control and decrease glycosylated haemoglobin (HbA1c) up to 2% through diet control (8-10). Coupled with this, appropriate dietary practices play a vital role in treating diabetes mellitus and, to some extent, prevent the complications of diabetes by improving risk factor profile. Strong body of evidence suggests that role of specific dietary factors remain uncertain; however, obesity and high intake of fat are associated with increased risk of diabetes (11-14). Despite the importance of diet in the management of T2DM, diabetic subjects are often unaware of the importance in ensuring glycaemic control (15). On the other hand, lack of dietary compliance is a major limiting factor in achieving glycaemic control in T2DM. Studies revealed that generally patients fail to adhere to dietary recommendations (16,17). Hence, the present study aimed at providing the profile of the factors associated with consumption of diabetic diet among T2DM subjects from western India as an impetus for further exploration of the sociocultural and subject-related factors affecting the outcomes of T2DM care that, in turn, will lead to redefine the diabetes control and prevention strategies in this region.

## MATERIALS AND METHODS

### Study setting

A hospital-based cross-sectional study was conducted during July 2010–November 2010 in Ahmedabad district of Gujarat state, India. Ahmedabad is the commercial hub of the Gujarat state with an approximate population of six million.

### Study population

The study population comprised diabetic subjects. We required them to be at least 40 years of age, have been diagnosed with T2DM for at least one year before enrollment for the study and, above all, it was a prerequisite for subjects to attend the Department of Diabetology, All India Institute of Diabetes and Research and Yash Diabetes Specialities Centre (Swasthya Hospital) during the study period.

### Sample-size and sampling method

A sample-size of 405 was obtained by using parameter estimation method with 5% precision around the point estimate (an expected diabetic diet consumption level of 50%) with 95% confidence level. The calculated minimum sample-size was inflated by 5% to account for anticipated non-response from subjects.

On an average, there are at least 40 known diabetes cases reporting to OPD, and the monthly load of known diabetics will be around 900-1,000. Hence, during the study period of five months, the cases will be around 4,500–5,000. The study subjects were selected through systematic random sampling strategy for 4,000 study subjects (on conservative side) who reported to the OPD. Selection of the first case was done from within the first 10 subjects (in our case, it was number 6), followed by adding 10 to the number till the required sample-size of 405 was achieved.

### Procedure for data collection

After recruitment of the subjects based on selection criteria and obtaining informed consent, the details of the study methodology were explained to them; a detailed history, including data on age, sex, education, occupation, smoking status, alcohol consumption, diabetic diet, method of cooking, and source of advice regarding diet, were recorded on a close-ended proforma. Diabetic diet was defined as a dietary adjustment for patients with diabetes mellitus intended to decrease the need of insulin or oral hypoglycaemic agents to avoid wide fluctuations in plasma glucose levels and to control weight by adjusting caloric and carbohydrate intake. Diabetic diet usually contains low-glycaemic index food, with similar amount of protein, complex carbohydrates, fibres, and unsaturated fatty acids as in food for general public. Diabetic diet was evaluated by dietary recall of the last 3 days, including timings, quantity of each meal and snacks, frequency of extra meal and food consumed from outside home, and the calculated average calorie consumed per day. All the subjects were also interviewed regarding history of hypertension. A general physical examination was also performed.

### Measurements

All anthropometric measurements were recorded using standardized procedures. Study subjects also underwent various clinical tests, such as blood tests for plasma glucose, glycosylated haemoglobin (HbA1c), and lipid levels. Blood samples were collected after ensuring 12 hours of overnight fasting. Total lipids, triglycerides (TG), and high-density lipoprotein-cholesterol (HDL-C) levels were estimated in serum, using kits (End Point Assay with Liquid Clearing Factor–LCF, Span Diagnostics Ltd. India). Low-density lipoprotein-cholesterol (LDL-C) was calculated using the Friedewald formula: LDL-C+TC-[HDL-C–(TG in mg/dL/5)] (18).

The current status of diabetes mellitus was measured using the criteria established by the American Diabetes Association (19), i.e. a medical record indicating either a fasting plasma glucose (FPG) level of >7.0 mmol/L or ≥126 mg/dL after a minimum 12-hour fasting, or 2-hour post-glucose level (oral glucose tolerance test) of >11.1 mmol/L or ≥200 mg/dL on more than one occasion, with symptoms of diabetes.

Blood pressure was recorded after the subjects had rested for at least ten minutes. The equipment was mercury sphygmomanometer (Diamond Deluxe BP apparatus, Pune, India). The machine was regularly inspected and validated. An appropriately-sized cuff (cuff bladder encircling at least 80% of the arm) was used for ensuring accuracy. It was applied on the right arm. The stethoscope bell was placed lightly over the brachial artery, and the blood pressure was recorded to the nearest 2 mmHg, reading from the top of the mercury meniscus. Systolic blood pressure (SBP) was recorded at the first appearance of two or more Korotkoff sounds and the disappearance of Korotkoff sound (onset of phase 5) was used for defining the diastolic blood pressure (DBP). Two readings were taken ten minutes apart, and mean of the two was considered the actual blood pressure. Hypertension was diagnosed based on the drug treatment for hypertension or if the blood pressure was >130/80 mmHg according to the Joint National Committee-7 (JNC-VII) criteria for diabetics (20,21). The study subjects were classified into two groups, based on the history of antihypertensive drugs. The study subjects in group one were defined as not taking drugs for hypertension, were without past history of hypertension with normal blood pressure at the time of study or were detected for the first time to have hypertension, and known cases of hypertension not taking antihypertensive drugs. In group two, the study subjects were defined as taking drugs for hypertension, were having either hypertension in control with drugs or uncontrolled blood pressure even with drugs.

Body mass index (BMI) values for Indians were defined according to the recommendations by Indian Council of Medical Research. A study subject was considered to be obese if BMI was ≥25 kg/m^2^ and overweight when BMI was 23-24.9 kg/m^2^ (22).

Glycosylated haemoglobin (HbA1c) was measured by the high-pressure liquid chromatography (HPLC) method, using the variant machine (BIORAD, Hercules, California, USA). Reference non-diabetic range is 4.0-6.0%. Control sera were included in each batch of samples analyzed. The criterion for glycaemic status was <7% (good control), 7-8% (suboptimal control), 8-9% (inadequate control), and >9% (uncontrolled) (23).

### Data analysis

Data were analyzed using the SPSS software (version 11.5). Means, standard deviations, and percentages were used for descriptive analysis. Student's *t*-test was used for testing the significance of differences between the mean values of two continuous variables. Univariate logistic regression analysis was conducted by comparing the outcome variable (consuming diabetic diet) with each independent variable of interest (age, sex, visiting dietician, level of education, occupation, glycaemic status, duration, and family history of diabetes), using odds ratio (OR) and their 95% confidence intervals (CI). Likelihood ratio test was used in estimating odds ratio and 95% CI for all associations of interest. Multivariate logistic regression analysis was performed to adjust for simultaneous effects of multiple factors or to control the effects of confounding factors on the outcome variable. The logistic regression model was used because the dependent variable was dichotomous (24)—either a diabetic subject was consuming diabetic diet or not consuming diabetic diet. The criteria for inclusion of factors in the multivariate analysis were: all variables from the univariate analysis with a p value of ≤0.1, along with all the variables of known biological importance. To assess the importance of each variable included in the model, Wald statistic for each variable was used. The parameters of the logistic regression model were estimated by the maximum likelihood method. The adjusted odds ratios (ORs) and their 95% confidence intervals (CIs) were computed using the estimates of parameters of final model. Selection of final model was based on parsimony, biological interpretability, and statistical significance. The probability (p) level of less than 0.05 was considered significant.

### Ethical approval

The Institutional Review Board of the All India Institute of Diabetes and Research reviewed and approved the study protocol and instrument.

## RESULTS

A sample of 405 diabetic subjects was enrolled. Of the total study population, 399 (98.5%) had T2DM, and 6 (1.5%) had type 1 diabetes mellitus. Hence, analysis was performed on 399 T2DM subjects. Of 399 T2DM, 65% were male, and 96% were literate. The study subjects were evenly distributed in four quartiles of age with mean of 53.16±7.95 years. Ninety-one percent (365/399) of the subjects were following the Hinduism religion (Table 1). The mean weight (kg) and height (cm) were (69.04±10.50) and (164.51±9.75) respectively. The mean duration of diabetes since diagnosis was 5.95±4.42 years.

### Risk and behaviour profile

The findings of the study showed that 140 (35%) had good glycaemic control (HbA1c <7%). The findings showed the subjects had a mean BMI of 25.57±4.05. Only 21% of the subjects were of normal weight; the majority were either overweight (BMI 23–24.99 kg/m^2^, 24%) or obese (BMI ≥25 kg/m^2^, 52%). About half (198/399) of the subjects had positive history of hypertension, and three-fourths (300/399) had positive family history of diabetes. Only 37% of the subjects were performing self-testing for blood sugar. The study also revealed that 95 (24%) of the subjects were smokers, and 32 (8%) were consuming alcohol (Table 1).

**Table 1.** Sociodemographic characteristics and profile of clinical and other associated factors of type 2 diabetic subjects from Ahmedabad, Western India (n=399)

Characteristics	Number (n=399)	Percentage*
Age (years)		
Up to 47	109	27
>47–52	98	25
>52–58	102	25
>58	90	23
Sex		
Male	259	65
Female	140	35
Marital status		
Never married	14	4
Ever married	385	96
Religion		
Hinduism	365	91
Islam	15	4
Christianity and others	19	5
Level of education		
No education	14	4
Primary school	84	21
Secondary school	176	44
College level	107	26
University level	14	4
Professional degree (CA, MBA, MBBS, etc.)	4	1
Occupation		
Govt. service	8	2
Professional	12	3
Private service	87	22
Business	116	29
Household work/retired	176	44
Type of diabetes		
T2DM	399	100
Mode of diagnosis of diabetes		
Symptomatic	373	93
At screening	23	6
Incidental	3	1
Duration of diabetes (years)		
1–2	105	26
>2–5	120	30
>5–9	90	23
>9	84	21
Glycaemic status (%)		
<7 (good control)	140	35
7–8 (suboptimal control)	120	30
>8–9 (inadequate control)	80	20
>9 (uncontrolled)	59	15
Family history of diabetes		
Positive	300	75
Negative	99	25
Body mass index (BMI) group		
Underweight (<18.5 kg/m^2^)	13	3
Normal (18.5-22.9 kg/m^2^)	83	21
Overweight (23.0–24.9 kg/m^2^)	95	24
Obese (≥25.0 kg/m^2^)	208	52
Self-monitoring blood sugar		
Yes	146	37
No	253	63
Hypertension		
Present	198	50
Not present	201	50
Smoker		
Yes	95	24
No	304	76
Alcohol consumption		
Yes	32	8
No	367	92

*All percentages rounded to whole numbers

There was a significant (p<0.05) difference between male and female subjects with respect to mean weight (male=71.101±9.809 kg, female=65.165±10.498 kg), height (male=169.83±6.875 cm, female=154.66±5.831 cm), and BMI (male=24.64±3.523, female=27.30±4.418) (Table 2).

**Table 2.** Characteristics of the study population, clinical and laboratory findings by sex among type 2 diabetic subjects from Ahmedabad, Western India (n=399)

Characteristics	Mean±SD	p value
	Male		Female
Age (years)	52.95±8.136	53.55±7.602	0.475
Weight (kg)	71.101±9.809	65.165±10.498	<0.001
Height (cm)	169.83±6.875	154.66±5.831	<0.001
Body mass index (kg/m^2^)	24.64±3.523	27.30±4.418	<0.001
Duration of diabetes (years)	6.027±4.353	5.828±4.558	0.669
Blood pressure without drugs (n=201)			
Systolic (mmHg)	130.45±14.076	132.39±14.639	0.350
Diastolic (mmHg)	83.15±7.261	83.17±8.166	0.986
Blood pressure with drugs (n=198)			
Systolic (mmHg)	135.34±16.845	134.93±15.936	0.861
Diastolic (mmHg)	83.14±9.469	84.10±8.207	0.598
Lipid profile (n=389)			
LDL cholesterol (mg/dL)	111.19±36.297	116.34±33.184	0.172
HDL cholesterol (mg/dL)	41.42±5.000	42.74±6.029	0.220
Triglycerides (mg/dL)	187.93±118.826	181.67±119.690	0.623
Total lipids (mg/dL)	714.46±167.240	711. 99±143.014	0.884

### Dietary practices

Majority (73%) of the subjects consuming diabetic diet were recommended by the family physicians/dieticians. However, only 39% reported that they had visited dietician since their diagnosis of diabetes, and only 2% reported counting calorie intake. Doctors/family physicians were reported to be the good source of advice regarding diet by 77% study population. The main method of cooking was: boiling and roasting (36%). Majority (88%) reported taking low fat or skimmed milk (Table 3).

Results of univariate analysis showed that visit to a dietician (OR=9.7, 95% CI=4.898–19.465), secondary level of education (OR=2.6, 95% CI=1.611–4.128), and low intake of fat (OR=2.6, 95% CI=1.385–4.760) are significantly associated with consumption of diabetic diet among T2DM subjects. Family history of diabetes is marginally associated with consumption of diabetic diet among T2DM subjects (Table 4). However, in univariate analysis, not a single factor was significantly associated with glycaemic status.

The final multivariate logistic regression model revealed that compared to the subjects not consuming diabetic diet, those who consumed diabetic diet were more likely to visit dietician (adjusted OR=10.6, 95% CI=5.124–21.816), consume low fat (adjusted OR=2.2, 95% CI=1.078–4.291), had higher level of education (adjusted OR=3.5, 95% CI=2.020–5.948), and have positive family history of diabetes (adjusted OR=1.8, 95% CI=0.996–3.094) (Table 5).

## DISCUSSION

Diabetes, literally a ‘sweet’ disease, is slowly but surely spreading around the world, and India today is home to one of the world's fastest-growing diabetic population (25). Hence, need for preventive actions through lifestyle modifications (diet control, physical exercise, etc.) would be widely given due importance (26).

The main factors observed in our study population were visit to dieticians, level of education, consumption of low fat/skimmed milk, and presence of family history of diabetes. Majority (88%) of the study subjects were consuming low fat/skimmed milk, which differ from a study by Al-Kaabi *et al*. (23). The reason for discrepancy may be that our study subjects were more likely to seek dietary advice from family physician/dietician. Diet planning is the mainstay in the self-management and control of T2DM (23). In the present study, 73% of the subjects were consuming diabetic diet, which differs from our previous report (27). This suggests that our study population was seriously considering the dietary advice. The main sources of dietary advice were: family physicians (77%) and dieticians (4%) who had a minimal role in dietary advice; the finding is consistent with those of previous studies (15,23). The possible explanation for this may be: easier access to family physicians than dieticians.

**Table 3.** Dietary practices among type 2 diabetic subjects from Ahmedabad, Western India (n=399)

Characteristics	Number (n=399)	Percentage*
Consuming diabetic diet		
Yes	290	73
No	109	27
Consuming low fat/skimmed milk		
Yes	351	88
No	48	12
Visited dietician since diagnosis of diabetes		
Yes	154	39
No	245	61
Counting daily calorie intake		
Yes	7	2
No	392	98
Methods of cooking		
Boiling and frying	119	30
Boiling and roasting	143	36
Roasting and frying	66	16
Boiling, frying, and roasting	71	18
Best source of advice regarding diet		
Self	51	13
Family member	18	4
Friend/colleague	4	1
Doctor/family physician	306	77
Dietician	15	4
Self, family member, and doctor	5	1
Management of diabetes through drugs with diet or physical activity		
Yes	342	86
No	57	14
Using drugs to control diabetes		
Yes	173	43
No	226	57
Following physical activities, recommendations by family physician		
Yes	215	54
No	184	46

*All percentages rounded to whole numbers

This study confirms our previous report regarding family history of diabetes among T2DM subjects (27). Result also showed that very few study subjects (14/399) were illiterate. This is expected because sample of this study was drawn from speciality hospital located in urban area.

Our findings for obesity (52%) among T2DM subjects are consistent with previous reports (28-31). Findings of this study also confirm the previous reports of low level of self-monitoring of blood sugar (32). This suggests that there might be a lack of awareness of its importance in relation to control of diabetes.

In this study, only 35% subjects had good glycaemic control, which is supported by reports from Holmström *et al.* (34%) and Al-Maskari *et al*. (38%) (33-34). However, this study could not demonstrate any significant association between diabetic diet and glycaemic control, despite there is high parcentage of study subjects consuming diabetic diet. This suggests that our study subjects might have started consuming diabetic diet recently, which has insignificant impact on glycaemic status in short duration.

### Limitations

The study has some weaknesses; only diet recall method was used for the last 3 days, and the study did not use any additional data on duration of diabetic diet consumption, which may either reduce or exaggerate the result of glycaemic control. This might be the reason behind insignificant association between diabetic diet and glycaemic control. Total reliance on the subjects regarding diabetic diet was another limitation. This is a hospital-based study from urban setup, which may not be representative of and applicable to general population. However, this could provide a reasonably precise and reliable estimate of factors associated with consumption of diabetic diet among T2DM subjects from western India. Such weaknesses may become a guide for future studies.

### Conclusions

This study revealed that majority (73%) of the subjects were consuming diabetic diet. The study also showed that visit to dieticians, level of education, consumption of low fat/skimmed milk, and presence of family history of diabetes were the main factors associated with consumption of diabetic diet. Self-monitoring of blood sugar is done by few subjects (37%).

**Table 4.** Univariate logistic analysis of the factors among hospital-based T2DM subjects from Ahmedabad, Western India (n=399)

Variable	Consuming diabetic diet		OR	(95% CI)	p value
	Yes (n=290)	No (n=109)			
Age (years)					
Up to 47	79	30	1.07	(0.575-1.989)	0.831
>47–52	74	24	1.25	(0.655-2.394)	0.496
>52–58	73	29	1.02	(0.546-1.914)	0.944
>58	64	26	1	-	
Sex					
Male	189	70	1.04	(0.658-1.652)	0.859
Female	101	39	1	-	
Visit to dieticians					
Yes	144	10	9.7	(4.898-19.465)	<0.001
No	146	99	1	-	
Level of education					
No education	6	8	0.48	(0.158-1.479)	0.203
Up to secondary	208	52	2.58	(1.611-4.128)	<0.001
Above secondary	76	49	1	-	
Occupation					
Housewifery/retired	133	43	1.32	(0.771-2.260)	0.312
Business	82	34	1.03	(0.579-1.829)	0.922
Service	75	32	1	-	
Duration of diabetes (years)					
1-2	69	36	0.81	(0.438-1.506)	0.509
>2-5	97	23	1.78	(0.931-3.431)	0.081
>5-9	65	25	1.10	(0.571-2.125)	0.773
>9	59	25	1	-	
Family history of diabetes					
Positive	224	76	1.47	(0.901-2.411)	0.123
Negative	66	33	1	-	
Consuming low fat/skimmed milk					
Yes	264	87	2.56	(1.385-4.760)	0.003
No	26	22	1	-	
Glycaemic status (%)					
<7	99	41	1	-	
≥7	191	68	1.16	(0.736-1.838)	0.517

OR=Odds ratio;

CI=Confidence interval

### Recommendations

Based on our findings we recommend the following:

- Visit to a dietician must be emphasized
- Frequent self-monitoring of blood sugar level needs to be taught and encouraged because it is associated with good glycaemic control.

**Table 5.** Multivariate logistic regression analysis of factors of consuming diabetic diet among hospital-based T2DM subjects from Ahmedabad, Western India (n=399)

Variable	AOR	95% CI	p value
Visit to dieticians			
Yes	10.6	5.124-21.816	<0.001
No	1	-	
Level of education			
No education	0.7	0.215-2.453	0.606
Up to secondary	3.5	2.020-5.948	<0.001
Above secondary	1	-	
Family history of diabetes			
Positive	1.8	0.996-3.094	0.051
Negative	1	-	
Consuming low fat/skimmed milk			
Yes	2.2	1.078-4.291	0.030
No	1	-	

AOR=Adjusted odds ratio;

CI=Confidence interval

Despite limitations, this study underlines the need for further investigation in India through longitudinal and cohort study designs to establish the association between consumption of diabetic diet and glycaemic control

## ACKNOWLEDGEMENTS

The study was supported by All India Institute of Diabetes and Research, Ahmedabad, India. The authors would like to express their sincere thanks to all the diabetic subjects who participated in the study. They would also like to thank Dr. W.Q. Shaikh for reviewing the manuscript.
